# Effects of Citric and Lactic Acid on the Reduction of Deoxynivalenol and Its Derivatives in Feeds

**DOI:** 10.3390/toxins8100285

**Published:** 2016-09-28

**Authors:** Elke Humer, Annegret Lucke, Hauke Harder, Barbara U. Metzler-Zebeli, Josef Böhm, Qendrim Zebeli

**Affiliations:** 1 Institute of Animal Nutrition and Functional Plant Compounds, Department for Farm Animals and Veterinary Public Health, University of Veterinary Medicine Vienna; Veterinaerplatz 1, Vienna 1210, Austria; elke.humer@vetmeduni.ac.at (E.H.); annegret.lucke@vetmeduni.ac.at (A.L.); hauke.harder@vetmeduni.ac.at (H.H.); josef.boehm@vetmeduni.ac.at (J.B.); 2 University Clinic for Swine, Department for Farm Animals and Veterinary Public Health, University of Veterinary Medicine Vienna, Veterinaerplatz 1, Vienna 1210, Austria; barbara.metzler@vetmeduni.ac.at

**Keywords:** citric acid, grain treatment, lactic acid, mycotoxin decontamination, trichothecenes

## Abstract

Exposure to mycotoxin-contaminated feeds represents a serious health risk. This has necessitated the need for the establishment of practical methods for mycotoxin decontamination. This study investigated the effects of citric acid (CA) and lactic acid (LA) on common trichothecene mycotoxins in feeds contaminated with *Fusarium* mycotoxins. Contaminated feed samples were processed either with 5% CA or 5% LA solutions in a ratio of 1:1.2 (*w*/*v*) for 5, 24, or 48 h, and analyzed for multiple mycotoxin metabolites using a liquid chromatography–tandem mass spectrometric method. The analyses showed that treating the feed with CA and LA lowered the concentration of deoxynivalenol (DON), whereby 5% LA lowered the original DON concentration in the contaminated feed samples by half, irrespective of the processing time. Similar lowering effects were observed for the concentrations of 15Ac-DON, 5-hydroxyculmorin, and sambucinol. The concentration of nivalenol was only lowered by the LA treatment. In contrast, CA and LA treatments showed no or only small effects on the concentration of several mycotoxins and their derivatives, including zearalenone, fumonisins, and culmorin. In conclusion, the present results indicate that the use of 5% solutions of LA and CA might reduce the concentration of common trichothecene mycotoxins, especially DON and its derivate 15Ac-DON. However, further research is required to determine the effect on overall toxicity and to identify the underlying mechanisms.

## 1. Introduction

The contamination of feeds with mycotoxins is a worldwide health concern in animal production, especially in the most sensitive livestock species, the pig [1]. Mycotoxins are toxic secondary metabolites produced by many species of fungi that grow on various agricultural commodities on the field site and during postharvest processes [2]. There is a vast diversity of mycotoxins that are produced by a plethora of different fungal genera. The major mycotoxin hazards regarding cereal preharvest in temperate regions (i.e., America, Europe, and Asia) are toxins of the genus *Fusarium* [3,4]. *Fusarium* mycotoxins, including deoxynivalenol (DON), nivalenol (NIV), T2, and HT2, constitute some of the most prevalent and harmful mycotoxins to animal productivity and health in temperate regions [4].

As mycotoxins are small and rather stable molecules, they are extremely difficult to remove or eradicate, and thus easily enter the feed chain while maintaining their toxic characteristics [5]. The prevention of mycotoxin formation includes pre- and post-harvest strategies [6]. As preventive measures in the field are often insufficient, additional procedures are necessary to decrease the mycotoxin contamination post-harvest. Mycotoxin reduction could be performed during feed processing using techniques that inactivate or destroy the mycotoxin [2]. Such decontamination processes should deactivate or even eliminate the mycotoxins without impairing the nutritive value and technological properties of the feed [7]. Supplementation of enzymes or microorganisms that are capable of degrading or modifying mycotoxins have recently been suggested to decrease or even eliminate their potential toxicity [4,8]. However, due to the lack of information about the effects of the converting reactions on the nutritive values of the feeds, the toxicity of transformation products, and on the safety towards animals, their application in practice has been limited [5]. Furthermore, a large range of chemicals has been shown to react with mycotoxins, thus converting them to less toxic compounds or even destroying them. These chemicals involve acids (e.g., hydrochloric acid, acetic acid, sulfuric acid), bases (e.g., ammonium, sodium hydroxide), oxidizing agents (e.g., hydrogen peroxide, ozone), reducing agents (e.g., bisulfites), chlorinating agents (e.g., chlorine), salts, gases, and miscellaneous reagents such as formaldehyde [3]. However, most of the methods using the aforementioned chemicals have limitations, due to being impractical, unsafe, and likely impairing the nutritive value as well as sensory and functional properties of the feed [9].

On the other hand, organic acids—such as citric acid (CA) or lactic acid (LA)—which are frequently used in food and feed conservation, as well as in feed processing, have been shown to improve the nutritional properties of feeds by promoting the degradation of anti-nutritive substances such as phytate, while improving the utilization of phytate-bound P. Moreover, an enrichment of cereals with healthy ingredients such as slowly degradable starch and fiber fractions has been observed, thus enhancing their health value in animal as well as human nutrition [10,11]. Previous research has also provided evidence for a certain detoxification property of some organic acids (including CA and LA) in aflatoxin- and ochratoxin-contaminated feeds raised under hot climatic conditions [9,12]. However, the effects of CA and LA treatment on the degradation of mycotoxins that predominate in temperate regions—especially trichothecenes and their derivatives—have not been investigated so far.

The hypothesis of this study was that treatment of experimentally contaminated feed with mild organic acids (i.e., LA and CA) is an effective method for the decontamination of mycotoxins predominating in temperate regions. Thus, the objective of this study was to characterize the potential of LA and CA to decontaminate trichothecene-contaminated cereal-based feeds soaked for different periods of time.

## 2. Results

**Figure 1:**
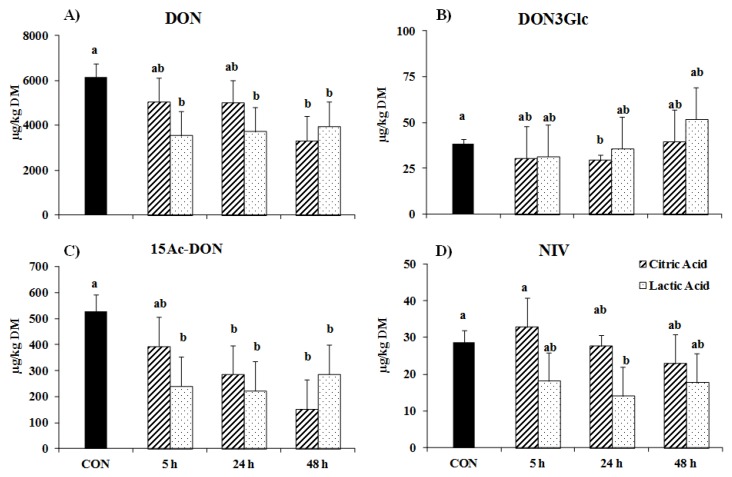
Concentration of (**A**) deoxynivalenol (DON), (**B**) DON3Glc, (**C**) 15Ac-DON, and (**D**) nivalenol (NIV) in feed samples either untreated (CON), or treated with citric acid (CA) or lactic acid (LA) at three different soaking times. Data are presented as least squares means ± standard errors. Statistically significant differences are indicated by differing lowercase letters (*p* ≤ 0.05).

The greatest reducing effect of the organic acid treatments in the soaked feed samples was found for DON and its derivate 15Ac-DON when compared to the control feed samples (*p* < 0.05, Figure 1). While the CA treatment reduced the initial concentration of 6.1 ± 0.58 mg DON per kg dry matter (DM) to 3.3 ± 1.10 mg per kg DM only after 48 h of soaking, a similar extent was already achieved after a soaking time of 5 h with the LA treatment (Figure 1A). The CA-treatment showed only a tendency for a lowering effect for DON3Glc compared to the control feed (*p* = 0.07, Figure 1B). Moreover, the LA treatment reduced the 15Ac-DON by about half at all soaking time points, whereas a similar reduction in 15Ac-DON was only achieved after soaking for 24 or 48 h with the CA treatment when compared to the control feed samples (*p* < 0.01, Figure 1C). Furthermore, the trichothecene NIV was reduced by on average 40% with the LA treatment of feed samples, with no differences among different soaking times. In contrast, the CA treatment showed no effect on this variable (Figure 1D).

The T2-toxin had an initial concentration of 1.4 ± 0.29 μg per kg DM and dropped below the detection limit after all acid treatments (Table 1).

**Figure 2:**
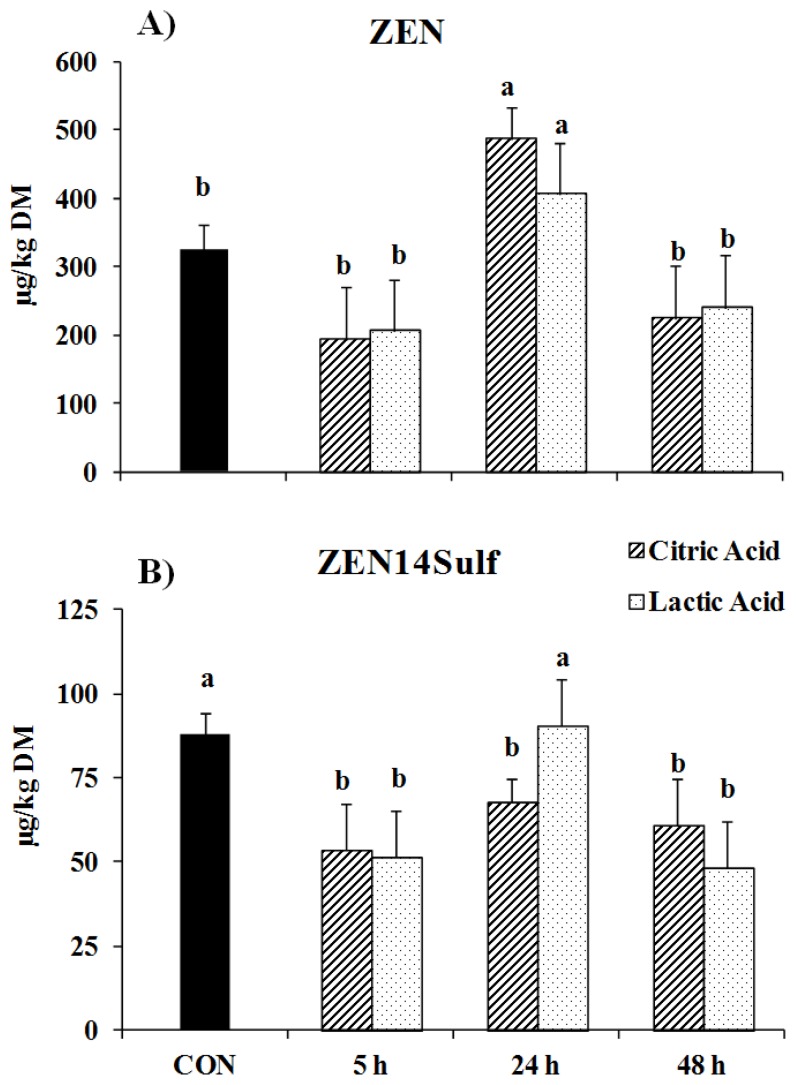
Concentrations of (**A**) zearalenone (ZEN) and (**B**) ZEN14Sulf in feed samples either untreated (CON) or treated with citric acid (CA) or lactic acid (LA) at three different soaking times. Data are presented as least squares means ± standard errors. Statistically significant differences are indicated by differing lowercase letters (*p* ≤ 0.05).

The average concentration of zearalenone (ZEN) in the contaminated feed samples of 0.3 ± 0.04 mg per kg DM was not decreased by the organic acid treatments (*p* = 0.86), but increased after 24 h of soaking (Figure 2A). By contrast, its derivate ZEN14Sulf was reduced by the CA treatments at all soaking times (*p* < 0.05, Figure 2B). Although the LA treatment lowered the ZEN14Sulf concentration at 5 h of soaking (*p* < 0.05), the ZEN14Sulf concentration increased again to similar concentrations as in the control feed after 24 h of soaking, and decreased (*p* < 0.05) again after 48 h of soaking with the LA solution.

Both organic acids affected the concentrations of the B-fumonisins (FBs) FB_1_ and FB_2_ (*p* < 0.05, Table 1). More specifically, the CA treatment resulted in an increased FB_1_ concentration after 24 and 48 h of soaking, whereas it caused an increased FB_2_ concentrations at all soaking times, with the greatest concentration after 48 h compared to the control feed samples. The LA treatment only led to an increased concentration of FB_1_ after 24 h of soaking, whereas the concentrations of FB_2_ in the LA-treated feeds did not differ from the initial value, regardless of the soaking time.

While for culmorin, only the LA treatment after 24 h of soaking showed a significant reduction, both organic acid treatments reduced or tended to reduce its derivatives 5-hydroxyculmorin and 15-hydroxyculmorin compared to the control feed samples (*p* ≤ 0.09). The CA treatment reduced both culmorin-derivatives only after soaking for 48 h, whereas the LA treatment achieved a similar reduction in 5-hydroxyculmorin at all soaking times (*p* < 0.01).

The concentrations of enniatin A, enniatin A_1_, enniatin B, and enniatin B_1_ were not affected by the acid treatments. However, processing with CA reduced enniatin B_2_ (*p* = 0.01).

Concentrations of sambucinol, decanolectrin, beauvericin, antibiotic Y, altersetin, infectopyron, nitropropionic acid, asperglaucide, asperphenamate, lotaustralin, noechinulin A, and tryptophol were lowered by the acid treatments, while the opposite effect was noticed for tenuazonic acid and rugulusovin (*p* ≤ 0.07).

Fusaric acid showed highest concentrations when the feed was treated for 24 h with LA, while after 48 h, both acid treatments enabled a reduction below the detection limit. Tentoxin was increased by the LA-treatment (*p* = 0.01), but not through soaking in CA. On the contrary, increased concentrations of fusarinolic acid and brevianamid F were measured only after the CA-treatment (*p* ≤ 0.07). The concentrations of the two bioactive cyclic dipeptides cyclo (L-Prol-L-Tyr) and cyclo(L-Prol-L-Val) were reduced by both treatments, but to a greater extent with LA compared to CA (*p* < 0.01). Apicidin was only reduced by the CA-treatments (*p* < 0.01). The acid treatments showed no effect on the concentrations of monilformin, aurofusarin, epiequisetin, equisetin, fusarin C, alternariol, alternariolmethylether, curvularin, or emodin.

## 3. Discussion

The main finding of this study was that the use of 5% solutions of LA and CA are able to reduce the concentration of common trichothecene mycotoxins, especially of DON, its derivate 15Ac-DON, and NIV. In contrast, the acid treatments showed limited effects on the concentration of several other mycotoxins and their derivatives, including ZEN, FBs, and culmorin. Thus, the findings of this study suggest that soaking contaminated feeds in mild organic acid solutions may offer a tool to reduce the mycotoxin load, especially that of trichothecenes.

Trichothecenes are the major toxic secondary metabolites produced by *Fusarium* species. They are globally distributed, even in the more extreme environments [13], and are the main pollutant in temperate regions, typically contaminating field crops, such as wheat, barley, oat, spelt, and maize [5,14]. Accordingly, T2 is the most abundant mycotoxin among type A trichothecenes in wheat, rye, and soybeans. This toxin has also been considered as one of the most acutely toxic mycotoxins among all trichothecenes [4,15]. In our study, T2 was detected but was comparatively low in the contaminated samples, whereas it dropped under the detection limit after both acid treatments at all soaking times. Thus, the decreasing effect of the LA- and CA-treatments on T2 deserves further investigation to elucidate the decontamination potential of the organic acid treatments, especially in feeds with higher concentrations of this toxin.

Among the type B trichothecenes, DON, DON3Glc, 15Ac-DON, and NIV were present in the contaminated feed samples in large amounts. Among these groups, DON represents the most prevalent mycotoxin in all kinds of cereals [4,16]. While the DON3Glc was resistant to the acid treatments, DON, 15Ac-DON, and NIV were reduced by both acid treatments, especially by the LA treatment. The reason why the acid treatments led to a reduction of 15Ac-DON but did not affect DON3Glc is most likely due to the hydrolysis of 15Ac-DON to DON. Indeed, it is known that DON3Glc is extremely resistant to acids (e.g., even at pH 0.7 obtained by treatment with 0.2 M HCl [17], a pH value that cannot be reached with the used mild organic acids in this study). On the other hand, acetylated forms of DON are extremely labile and hydrolyze rapidly to DON under slight acidic conditions or in vivo [18]. Thus, the reduction in 15Ac-DON cannot be designated as mitigation per se, but rather an interconversion. However, the overall DON concentration decreased due to both acid treatments. While it seems that feed has to be soaked in CA for at least 24 h to reach a substantial reduction of most of the detected trichothecenes, similar effects can be achieved with LA even after a short soaking period of 5 h. Thus, treatments of feeds with LA might offer an effective method to reduce the concentration of those mycotoxins in the feed, even after short soaking procedures. Nevertheless, an assessment of the overall toxicity has to be conducted in further studies to confirm the significance of the present findings.

Besides trichothecenes, ZEN represents a further major mycotoxin compound produced by various *Fusarium* species. Although they are commonly found in maize, several *Fusarium* species are also found in other crops, such as barley, wheat, sorghum, rye, and even soybeans [19,20]. Among the conjugates that can be formed from ZEN, we detected ZEN14Sulf in the contaminated samples, which is a common metabolite produced by sulfonation processes [21]. Results of this study showed an increase in the ZEN concentration in feed samples treated with acids for 24 h. One possible reason might be the release of masked ZEN by the acid treatments, but this needs to be clarified with further studies. In contrast, its metabolite ZEN14Sulf was reduced by the acid treatments. Thus, it can be concluded that both acid treatments are suitable for decontamination of the ZEN metabolite ZEN14Sulf, as long as the soaking time is either quite short (i.e., 5 h) or almost 48 h. Although this metabolite has been shown to possess a reduced oestrogenic toxicity [8,21], it has not yet been clarified whether the sulfonation leads to an effective detoxification, as it is possible that a hydrolysis of this conjugate could occur in the digestive tract [7]. Therefore, the benefit in the reduction of this metabolite by organic acid treatment has to be studied in further research.

Further prevalent mycotoxins produced by *Fusarium* species are culmorin and hydroxyculmorins (i.e., 5-hydroxyculmorin and 15-hydroxyculmorin), which are mainly produced by *Fusarium culmorum*. Those toxins have been detected in similar concentrations as DON in naturally contaminated grain [22]. In the present study, both acid treatments were effective in reducing these mycotoxins, whereby a higher effect was achieved after soaking in LA. Although low toxicity has been reported for culmorin and hydroxyculmorins using in vitro assays [23], they may contribute to enhancing the toxicity of DON [24]. Thus, the reducing effect of the acid treatments on the concentrations of trichothecene might even be enhanced further through their lowering effects on culmorins.

In addition to culmorins, a major metabolite produced by *Fusarium culmorum* is sambucinol [25]. In accordance with the effects observed for culmorin and hydroxyculmorins, both acid treatments were effective in reducing sambucinol concentrations by about half of the initial concentration. However, as the role of this metabolite is unknown [26], the nutritional significance of this reduction needs to be clarified in further studies. Nevertheless, one has to keep in mind that synergistic interaction among toxins produced by *Fusarium culmorum* and *Fusarium graminearum* might occur [27,28].

As stated above, aside from trichothecenes and ZEN, *Fusarium* species also produce FBs [18]. In general, FB_1_ and FB_2_ were detected in the contaminated samples, but were far below the guidance levels recommended by the European Union Commission [5]. Overall, the acid treatments failed to reduce FB contents. On the contrary, the CA treatments especially enriched the FBs in feeds samples, while LA only negatively affected FB_1_ after a soaking time of 24 h. Further research is required to identify the mechanisms behind this.

In addition to the aforementioned most important classes of mycotoxins produced by *Fusarium* species, *Fusarium* genera also produce emerging mycotoxins, such as enniatins, beauvericin, and moniliformin, which are less studied as they are more recently discovered [4]. Among the detected enniatins, enniatin A, A_1_, B, and B_1_ remained unaffected by the treatments, whereas the CA treatment decreased the concentration of enniatin B_2_. While beauvericin showed a trend towards a reduction after the acid treatments, monilformin concentrations were not affected by the treatments. Although these mycotoxins have been considered as less important for some years due to their low probability of acute toxicity, they possess a high prevalence in feed products [2,29]. Thus, a potential effect of acid treatments on the concentrations of those toxins could be of significance, but needs to be clarified with further studies.

Among the *Alternaria* metabolites, tentoxin and tenuazonic acid were found in higher concentrations in the LA-treated feeds and in LA- as well as CA-treated feeds, respectively, whereby only the latter is of toxicological concern [30]. On the contrary, altersetin was reduced by both acid treatments. However, the nutritional significance of this reduction remains unknown [31]. Furthermore, there are few data available on the toxicity of several other mycotoxins detected in the present study. Therefore, effects of changes in the concentrations of some of those toxins on animal health remain unclear. However, one has to keep in mind that the toxicological impact of mycotoxin mixtures is so far largely unknown [2].

To the best of our knowledge, this is the first study investigating the effect of treatments with organic acids on the detoxification of mycotoxins produced mainly by *Fusarium* species, as the few previous studies evaluating the effect of acid treatments solely focused on aflatoxins, and to a lesser extent on ochratoxins. Jalili et al. [32], for instance, evaluated the effect of 18 different chemicals—including CA, among other acidic compounds, alkaline compounds, and salts—in 2% concentrations of each chemical on the reduction of aflatoxins and ochratoxins in pepper. Although almost all of the applied chemicals showed a significant degree of mycotoxin reduction, the acidic compounds showed a weaker effect compared to the other chemicals (i.e., NaOH). However, as the alkaline treatments caused undesirable effects on the products, their application does not appear to be of practical significance. In contrast with this finding, Mendez-Albores et al. [33] observed a 97% degradation of the initial concentration of aflatoxin-contaminated maize or even a complete removal (depending on the initial concentration) through treatments with aqueous CA. Interestingly, the authors also demonstrated that the acidified samples were effective in reducing negative side effects of aflatoxins on toxicity, mutagenicity, and carcinogenicity without compromising the nutritional and organoleptic quality of the feed. Therefore, these treatment might offer a promising method to eliminate aflatoxin from food commodities, both in terms of efficacy as well as safety [9].

However, only a few studies report on the mode of action for the reducing effect of acid treatments on aflatoxin and ochratoxin concentrations. The reducing effect on aflatoxins has been reported to be due to the acid-catalysed addition of water to the vinyl ether double bond of several aflatoxins (i.e., AFB_1_ and AFG_1_) to convert them to their “hemiacetal” [32], whereas the mode of action for the decrease in ochratoxin has been assumed to be based on its conversion to phenylalanine and a lactone acid [34]. However, Jalili et al. [32] pointed out that although acidic solutions are able to destroy mycotoxins, the obtained degradation products might be unstable and tend to convert to their parent products.

Besides these few reports, the mechanisms behind the detoxifying effects on mycotoxins primarily produced by *Fusarium* species are so far unknown and thus require further investigation. Moreover, further studies should be carried out to identify possible byproducts resulting from the acid treatments, and toxicity tests on those products should be conducted. With appropriate test results, treating feeds with LA or CA might be a promising procedure for the detoxification of trichothecenes and their respective derivatives.

## 4. Conclusions

Taken together, processing contaminated feed samples with 5% CA and LA lowered the concentration of common mycotoxins, especially DON and its derivative 15Ac-DON and NIV. Treatment of the feed with LA resulted in a noticeable reduction of several mycotoxins after 5 h of soaking, whereas CA required more time to decontaminate certain mycotoxins. However, the underlying mechanisms remain unclear. Further research is warranted to determine their significance in animal trials.

## 5. Materials and Methods

### 5.1. Processing Procedure of Feed Samples with Organic Acids

In the present experiment, we used a cereal-based ground compound feed experimentally contaminated with *Fusarium* mycotoxins, whereby the average concentration of main trichothecene mycotoxins was 6.1 ± 0.58 mg DON, 5.3 ± 0.64 mg aurofusarin, 0.8 ± 0.20 mg fusarin C, and 0.3 ± 0.05 mg ZEN per kg DM. Processing method consisted of soaking 50 g of randomly taken contaminated feed samples in a solution containing either 5% CA (99.5% *w*/*w*, Solan Kraftfutterwerk Schmalwieser GmbH, Bachmanning, Austria) or 5% LA (DL-lactate, 80% *w*/*w*, Brenntag CEE GmbH, Vienna, Austria) at room temperature (22 °C). The detailed soaking procedure is reported elsewhere [35,36,37]. The ratio of feed to soaking solution containing either CA or LA was 1:1.2 (*w*/*v*), and the contaminated feed samples were soaked for either 5 (*n* = 3/acid), 24 (*n* = 6/acid), or 48 (*n* = 3/acid) h to evaluate a potential soaking time effect on the decontamination of the mycotoxins. After the processing procedure, the samples were immediately stored at −20 °C until analysis. The untreated contaminated feed samples (*n* = 12) that served as control treatment were also frozen and stored at −20 °C until analysis. All data were corrected for DM content of the feed.

### 5.2. Multi-Mycotoxin LC-MS/MS Analysis

The analysis of mycotoxins was carried out at the Center for Analytical Chemistry, Department of Agrobiotechnology (IFA-Tulln, Tulln, Austria) using a liquid chromatography-tandem mass spectrometric (LC-MS/MS) method for multiple mycotoxin metabolites, as described recently [38]. In brief, feed samples were thawed, and 5 g of the representative sub-samples were extracted using 20 mL of a mixture of acetonitrile:water:acetic acid (79:20:1, *v*/*v*/*v*). Thereafter, the samples were centrifuged, diluted 1:1 (*v*/*v*), and injected as described in detail by Sulyok et al. [39]. The LC-MS/MS determination of the mycotoxins was conducted with a QTrap 5500 LC-MS/MS System (Applied Biosystems, Foster City, CA, USA) equipped with TurboIonSpray electrospray ionization (ESI) source and a 1290 Series HPLC System (Agilent, Waldbronn, Germany). A Gemini^®^ C18-column (150 × 4.6 mm i.d., 5 μm particle size), equipped with a C18 (4 × 3 mm i.d.) security guard cartridge (Phenomenex, Torrance, CA, USA) was used for chromatographic separation at 25 °C. The ESI-MS/MS was performed in the scheduled multiple reaction monitoring (MRM) mode, both in positive and negative polarities in two separate chromatographic runs per sample by scanning two fragmentation reactions per analyte. Quantification of the fungal metabolites was carried out by external calibration, using a multi-component standard preparation from authentic standards. Details relating to spiking, recoveries, and further LC-MS/MS parameters are reported elsewhere [38].

The accuracy of the method is verified continuously for mycotoxins subject to regulatory limits by participation in proficiency testing schemes organized by CODA-CERVA, the Belgian National Reference Laboratory for Mycotoxins in Food and Feed [40], and CNR, the Institute of Sciences of Food Production, National Research Council of Italy [41].

### 5.3. Statistical Analysis

Data were processed by ANOVA using the MIXED procedure of SAS (SAS Institute Inc., Cary, NC, USA, version 9.2). The model accounted for the fixed effect of treatment and time as well as for the random effect of replicate. Comparisons among treatments were evaluated by the probability of difference (pdiff) option, and degrees of freedom were estimated with the method of Kenward–Roger. Furthermore, the significance of the overall effect of the acid treatment was tested using a linear contrast involving the average of the two acid groups vs. control (CON). Moreover, to assess the effect of LA- and CA-treatment separately, linear contrasts (CON vs. LA and CON vs. CA, respectively) were also performed. The significance level was set at *p* ≤ 0.05, and a trend was considered at 0.05 < *p* ≤ 0.10 level.

## Figures and Tables

**Table 1:** Concentration of mycotoxins (μg/kg dry matter, DM) found in experimentally contaminated feed samples either untreated (CON) or treated with 5% citric acid (CA) or 5% lactic acid (LA) as an average of the three soaking times (i.e., 5, 24, and 48 h).

Variable	Treatment				*p*-Value (Contrast)		
	CON	CA	LA	SEM	CON vs. LA	CON vs.CA	CON vs. LA + CA
T2	1.4	n.d.	n.d.	0.29	-	-	-
FB_1_	35.6 ^b^	58.0 ^a^	62.4 ^a^	5.08	<0.01	<0.01	<0.01
FB_2_	19.2 ^b^	25.2 ^a^	21.1 ^b^	1.21	<0.01	0.34	0.02
Culmorin	127.5 ^a^	116.5 ^ab^	103.7 ^b^	10.09	0.14	0.30	0.08
5-Hydroxyculmorin	740.4 ^a^	684.5 ^ab^	474.4 ^b^	20.83	<0.01	0.36	0.03
15-Hydroxyculmorin	314.4 ^a^	278.9 ^a^	259.0 ^b^	9.20	0.07	0.21	0.09
Sambucinol	179.0 ^a^	105.0 ^b^	92.9 ^b^	16.91	<0.01	<0.01	<0.01
Decalonectrin	87.0 ^a^	69.4 ^ab^	60.1 ^b^	10.09	0.22	0.07	0.08
Enniatin A	1.0	0.9	0.8	0.08	0.82	0.20	0.38
Enniatin A_1_	11.1	10.4	10.1	0.89	0.44	0.59	0.42
Enniatin B	40.1	34.1	50.7	4.61	0.68	0.37	0.12
Enniatin B_1_	23.5	19.5	20.5	2.21	0.24	0.37	0.24
Enniatin B_2_	1.2 ^a^	0.8 ^b^	1.2 ^a^	0.11	0.01	0.66	0.08
Moniliformin	10.1	10.1	16.4	2.27	0.99	0.11	0.35
Beauvericin	10.5 ^a^	8.5 ^b^	8.7 ^ab^	0.76	0.08	0.12	0.06
Aurofusarin	5306	5039	4562	642.5	0.77	0.42	0.53
Antibiotic Y	71.1 ^a^	30.4 ^b^	27.9 ^a^	6.50	<0.01	<0.01	<0.01
Apicidin	2.3 ^a^	1.7 ^b^	2.2 ^a^	0.10	<0.01	0.84	0.04
Epiequisetin	0.7	0.7	0.7	0.07	0.38	0.85	0.53
Equisetin	4.4	3.8	4.4	0.67	0.53	0.99	0.71
Fusaric acid	46.0 ^b^	40.1 ^b^	102.0 ^a^	24.78	0.84	0.08	0.33
Fusarin C	810.4	876.9	510.9	200.5	0.82	0.30	0.64
Fusarinolic acid	16.0 ^b^	61.1 ^a^	34.7 ^b^	10.99	0.01	0.25	0.03
Tenuazonic acid	65.5 ^b^	77.6 ^ab^	81.6 ^a^	6.06	0.17	0.08	0.07
Alternariol	17.1	15.9	15.0	0.89	0.36	0.13	0.17
Alternariolmethylether	7.6	6.8	8.7	0.71	0.47	0.27	0.83
Altersetin	54.7 ^a^	39.4 ^b^	30.8 ^b^	4.12	0.02	<0.01	<0.01
Tentoxin	0.4 ^b^	0.3 ^b^	0.9 ^a^	0.12	0.53	0.01	0.22
Infectopyron	352.0 ^a^	291.7 ^b^	298.3 ^b^	9.58	<0.01	<0.01	<0.01
Nitropropionic acid	52.4 ^a^	28.1 ^c^	41.2 ^b^	1.82	<0.01	<0.01	<0.01
Curvularin	5.7	6.7	7.4	1.33	0.65	0.44	0.49
Asperglaucide	8.9 ^a^	5.8 ^b^	6.3 ^b^	0.30	<0.01	<0.01	<0.01
Asperphenamate	2.7 ^a^	2.0 ^b^	2.1 ^b^	0.14	<0.01	<0.01	<0.01
Brevianamid F	32.2 ^b^	36.4 ^a^	26.6 ^b^	1.40	0.12	0.07	0.72
Cyclo(L-Pro-L-Tyr)	205.5 ^a^	139.4 ^b^	120.1 ^c^	10.11	<0.01	<0.01	<0.01
Cyclo(L-Pro-L-Val)	142.1 ^a^	125.3 ^b^	112.7 ^c^	3.39	<0.01	<0.01	<0.01
Emodin	5.3	4.9	3.8	0.78	0.74	0.19	0.34
Lotaustralin	44.8 ^a^	36.5 ^b^	38.1 ^ab^	3.04	0.12	0.02	0.04
Neoechinulin A	9.9 ^a^	5.2 ^b^	4.7 ^b^	0.44	<0.01	<0.01	<0.01
Rugulusovin	5.2 ^b^	7.5 ^a^	8.0 ^a^	0.60	<0.01	0.01	<0.01
Tryptophol	197.8 ^a^	175.3 ^b^	188.6 ^ab^	5.81	0.01	0.27	0.04

^a,b,c^ indicate differences at *p* < 0.05; n.d. = not detectable (below detection limit).

## References

[1] Ghareeb K., Awad W.A., Böhm J., Zebeli Q. (2015). Impacts of the feed contaminant deoxynivalenol on the intestine of monogastric animals: Poultry and swine. J. Appl. Toxicol..

[2] Marin S., Ramos A.J., Cano-Sancho G., Sanchis V. (2013). Mycotoxins: Occurrence, toxicology, and exposure assessment. Food Chem. Toxicol..

[3] Kabak B., Dobson A., Var I. (2006). Strategies to prevent mycotoxin contamination of food and animal feed: A review. Crit. Rev. Food Sci. Nutr..

[4] Escrivá L., Font G., Manyes L. (2015). In vivo toxicity studies of *Fusarium* mycotoxins in the last decade: A review. Food Chem. Toxicol..

[5] Boudergue C., Burel C., Dragacci S., Favrot M.C., Fremy J.M., Massimi C., Pringent P., Debongnie P., Pussemier L., Boudra H. (2009). Review of Mycotoxin-Detoxifying Agents Used as Feed Additives: Mode of Action, Efficacy and Feed/Food Safety.

[6] Bennett J., Klich M. (2003). Mycotoxins. Clin. Microbiol. Rev..

[7] Jard G., Liboz T., Mathieu F., Guyonvarch A., Lebrihi A. (2011). Review of mycotoxin reduction in food and feed: From prevention in the field to detoxification by adsorption or transformation. Food Addit. Contam..

[8] Jard G., Liboz T., Mathieu F., Guyonvarch A., Andre F., Delaforge M., Lebrihi A. (2010). Transformation of zearalenone to zearalenone-sulfate by *Aspergillus* spp.. World Mycotoxin J..

[9] Mendez-Albores A., Del Rio-Garcia J., Moreno-Martinez E. (2007). Decontamination of aflatoxin duckling feed with aqueous citric acid treatment. Anim. Feed Sci. Technol..

[10] Khol-Parisini A., Humer E., Sizmaz Ö., Abdel-Raheem S., Gruber L., Gasteiner J., Zebeli Q. (2015). Ruminal disappearance of phosphorus and starch, reticuloruminal pH and total tract nutrient digestibility in dairy cows fed diets differing in grain processing. Anim. Feed Sci. Technol..

[11] Metzler-Zebeli B.U., Deckardt K., Schollenberger M., Rodehutscord M., Zebeli Q. (2014). Lactic acid and thermal treatments trigger the hydrolysis of *Myo*-Inositol Phosphates and modify the abundance of lower *Myo*-Inositol Phosphates in barley (*Hordeum vulgare* L.). PLoS ONE.

[12] Mendez-Albores A., Martinez-Bustos F., Gaytan-Martinez M., Moreno-Martinez E. (2008). Effect of lactic and citric acid on the stability of B-aflatoxins in extrusion-cooked sorghum. Lett. Appl. Microbiol..

[13] Nelson P., Dignani M., Anaissie E. (1994). Taxonomy, biology and clinical aspects of *Fusarium* species. Clin. Microbiol. Rev..

[14] Eriksen G., Pettersson H., Lundh T. (2004). Comparative cytotoxicity of deoxynivalenol, nivalenol, their acetylated derivatives and de-epoxy metabolites. Food Chem. Toxicol..

[15] Seeboth J., Solinhac R., Oswald I., Guzylack-Piriou L. (2012). The fungal T-2 toxin alters the activation of primary macrophages induced by TLR-agonists resulting in a decrease of the inflammatory response in the pig. Vet. Res..

[16] Summerell B., Leslie J. (2011). Fifty years of *Fusarium*: How could nine species have ever been enough?. Fungal Divers..

[17] Berthiller F., Krska R., Domig K.J., Kneifel W., Juge N., Schuhmacher R., Adam G. (2011). Hydrolytic fate of deoxynivalenol-3-glucoside during digestion. Toxicol. Lett..

[18] World Health Organization (WHO) (2011). Evaluation of Certain Contaminants in Food. Seventy-Second Report of the Joint FAO/WHO Expert Comitee on Food Additives.

[19] Juan C., Ritieni A., Manes J. (2012). Determination of trichothecenes and zearalenones in grain cereal, flour and bread by liquid chromatography tandem mass spectrometry. Food Chem..

[20] European Food Safety Authority (ESFA) (2011). Scientific Opinion on the risks for public health related to the presence of zearalenone in food. EFSA J..

[21] Plasencia J., Mirocha C. (1991). Isolation and characterization of zearalenone sulfate produced by *Fusarium* spp.. Appl. Environ. Microbiol..

[22] Scarpino V., Reyneri A., Sulyok M., Krska R., Blandino M. (2015). Effect of fungicide application to control *Fusarium* head blight and 20 *Fusarium* and *Alternaria* mycotoxins in winter wheat (*Triticum aestivum* L.). World Mycotoxin J..

[23] Pedersen P.B., Miller J.D. (1999). The fungal metabolite culmorin and related compounds. Nat. Toxins.

[24] Ghebremeskel M., Langseth W. (2001). The occurrence of culmorin and hydroxy-culmorins in cereals. Mycopathologia.

[25] Zamir L., Nikolakakis A., Huang L., St-Pierre P., Sauriol F., Sparace S., Mamer O. (1999). Biosynthesis of 3-acetyldeoxynivalenol and sambucinol—Identification of the two oxygenation steps after trichodiene. J. Biol. Chem..

[26] Blackwell B.A., Seguin C., Overy D. (2012). Trichothecenes and other secondary metabolites from *Fusarium graminearum*—is it just about deoxynivalenol?. Can. J. Plant Pathol..

[27] Rotter R.G., Thompson B.K., Trenholm H.L., Prelusky D.B., Hartin K.E., Miller J.D. (1992). A preliminary examinatino of potential interactions between deoxynivalenol (DON) and other selected *Fusarium* mycotoxins in growing pigs. Can. J. Anim. Sci..

[28] Dowd P., Miller J., Greenhalgh R. (1989). Toxicity and interactions of some *Fusarium graminearum* metabolites to caterpillars. Mycologia.

[29] Gutleb A., Morrison E., Murk A. (2002). Cytotoxicity assays for mycotoxins produced by *Fusarium* strains: A review. Environ. Toxicol. Pharmacol..

[30] Streit E., Schwab C., Sulyok M., Naehrer K., Krska R., Schatzmayr G. (2013). Multi-mycotoxin screening reveals the occurrence of 139 different secondary metabolites in feed and feed ingredients. Toxins.

[31] Hellwig V., Grothe T., Mayer-Bartschmid A., Endermann R., Geschke F.U., Henkel T., Stadler M. (2002). Altersetin, a new antibiotic from cultures of endophytic *Alternaria* spp. taxonomy, fermentation, isolation, structure elucidation and biological activities. J. Antibiot..

[32] Jalili M., Jinap S., Son R. (2011). The effect of chemical treatment on reduction of aflatoxins and ochratoxin A in black and white pepper during washing. Food Addit. Contam..

[33] Mendez-Albores A., Arambula-Villa G., Loarea-Pina M., Castano-Tostado E., Moreno-Martinez E. (2005). Safety and efficacy evaluation of aqueous citric acid to degrade B-aflatoxins in maize. Food Chem. Toxicol..

[34] Van der Merwe K.J., Steyn P.S., Fourie L. (1965). Mycotoxins. II. The constitution of ochratoxins A, B, and C, metabolites of *Aspergillus ochraceus wilh*. J. Chem. Soc..

[35] Harder H., Khol-Parisini A., Zebeli Q. (2014). Treatments with organic acids and pullulanase differently affect resistant starch and fiber composition in flour of various barley genotypes (*Hordeum vulgare* L.). Starch-Stärke.

[36] Harder H., Khol-Parisini A., Zebeli Q. (2015). Modulation of resistant starch and nutrient composition of barley grain using organic acids and thermal cycling treatments. Starch-Stärke.

[37] Harder H., Khol-Parisini A., Metzler-Zebeli B.U., Klevenhusen F., Zebeli Q. (2015). Treatment of grain with organic acids at 2 different dietary phosphours levels modulates ruminal microbial communitiy structure and fermentation patterns in vitro. J. Dairy Sci..

[38] Diaz G., Krska R., Sulyok M. (2015). Mycotoxins and cyanogenic glycosides in staple foods of three indigenous people of the Colombian Amazon. Food Addit. Contam..

[39] Sulyok M., Berthiller F., Krska R., Schuhmacher R. (2006). Development and validation of a liquid chromatography/tandem mass spectrometric method for the determination of 39 mycotoxins in wheat and maize. Rapid Commun. Mass Spectrom..

[40] Debongnie P., Tangni E., Callebaut A. (2015). Report on the 2014 Proficiency Test for the Determination of 19 Mycotoxins and 4 Sums of Mycotoxins in Rye Flour.

[41] De Girolamo A., Ciasca B., Stroka J., Bratinova S., Visconti A., Lattanzio V.M.T. (2015). Report of the 2014 Proficiency Test for LC-MS(MS) Multi-Mycotoxin Methods, Determination of DON, FB1, FB2, ZEA, T-2, HT-2, OTA, AFB1, AFG1, AFB2, AFG2 in Maize and Determination of DON, ZEA, T-2, HT-2, OTA in Wheat.

